# Expression of yeast deubiquitination enzyme UBP1 analogues in *E. coli*

**DOI:** 10.1186/1475-2859-4-17

**Published:** 2005-05-30

**Authors:** Anna Wojtowicz, Anna Mazurkiewicz-Pisarek, Grazyna Plucienniczak, Diana Mikiewicz-Sygula, Luiza Chojnacka, Natalia Lukasiewicz, Andrzej Plucienniczak

**Affiliations:** 1 Institute of Biotechnology and Antibiotics, Staroscinska 5,02-516 Warsaw, Poland

## Abstract

**Background:**

It has been shown that proteins fused to ubiquitin undergo greater expression in *E. coli *and are easier to purify and renaturate than nonhybrid foreign proteins. However, there is no commercial source of large quantities of specific deubiquitinating proteases. This is the reason why hybrid proteins containing ubiquitin at their N-end cannot be used in large scale biotechnological processes.

**Results and Conclusion:**

We have described the synthesis of the yeast deubiquination enzyme UBP1 muteins in *E. coli*. We have shown that an efficient overproduction of the enzyme in *E. coli *may be achieved after the introduction of several changes in the nucleotide sequence encoding *UBP1*. One of the conditions of an effective synthesis of the UBP1 muteins is the removal of the 5'-end sequence encoding the transmembrane region of the enzyme. The obtained variants of the enzyme may be successfully used for processing large amounts of hybrid proteins comprising ubiquitin or tagged ubiquitin at their N-ends.

## Background

Ubiquitin is composed of 76 amino-acid residues with a total molecular mass of 8.6 kDa. This protein is an element of the universal protein modification in eukaryots called ubiquitination, a phenomenon which does not occur in bacteria. In spite of that, it has been shown that proteins fused to ubiquitin undergo greater expression in *E. coli *and are easier to purify and renaturate than nonhybrid foreign proteins [1]. However, to take advantage of these properties of hybrid proteins in technological processes, large amounts of proteases for cleaving specifically ubiquitin from those proteins are necessary. Protease UBP1, an enzyme found in the yeast *Saccharomyces cerevisiae*, is a candidate for becoming such a tool. The enzyme was described in 1991 [2] and is the subject of a patent application [3]. UBP1 is a cysteine protease which cleaves ubiquitin from protein fused to its C-end. Its activity and culture conditions in *E. coli *have been described [2], but the problem of a larger and more efficient production of the enzyme remains unsolved and for that reason technical applications of the inventions mentioned in [3] have not been possible.

The aim of this work was to obtain an expression system for an efficient synthesis of UBP1 protease variants that would be useful in industrial processes.

## Results and Discussion

**Figure 1:**
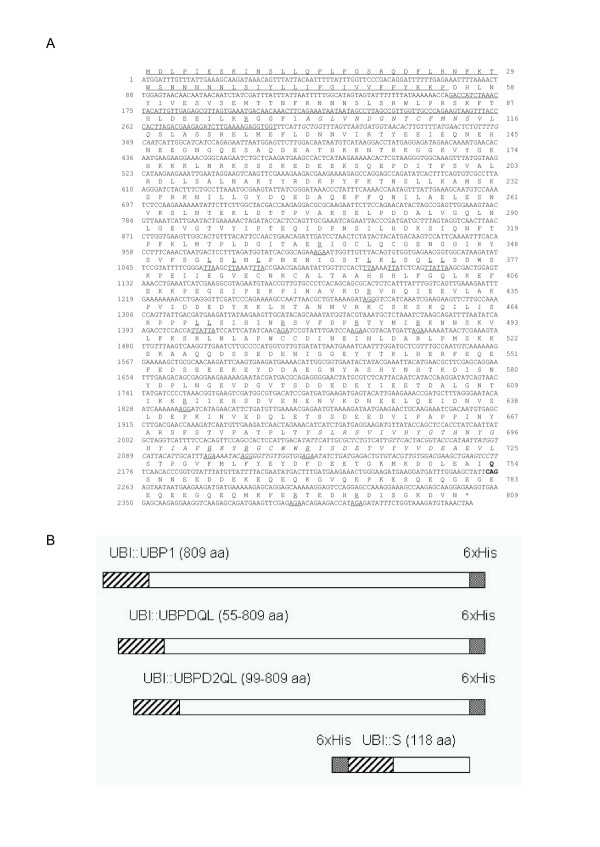
**Nucleotide and amino-acid sequences of the yeast *UBP1 *gene and UBP1 protease [2]**. A) The nucleotide residues are numbered on the left, and amino acid residues are numbered on the right of the figure. The underlined AGA or AGG arginine and TTA leucine codons were changed for CGC or CGT and CTG triplets, respectively. The CAG triplet (shown in bold) at positions 2260–2262 was changed to CTG one (Q754L mutation). The amino-acids forming active centre is shown in italics [4]. For the construction of *UBI::UBPDQL *or *UBI::UBPD2QL *coding sequences the first 162 bp or 294 bp which were removed are overlined or underlined, respectively. The 3'part of the *UBP1 *gene (positions: 2074–2430) was used to construct the S protein, the truncated hybrid protein which was used to determine the UBP1 variants' activity. B) Schematic structure of UBI::UBP1 construction variants.  – ubiquitin, □ – UBP1,  – 6xHis.

We were not able to obtain an efficient expression of the full coding sequence of *UBP1*. This is in agreement with the results of Tobias and Varshavsky [2], who were able to detect UBP1 in *E. coli *only after an immunoblot analysis. A possible reason for this situation might be the presence of a transmembrane region in the N-end part of the protein, which was discovered after a computer analysis using the TMHMM v. 2.0 software package (CBS, Denmark) [4]. The region encompasses amino-acids 34–51 (Figure 1). Because of that, we decided to prepare truncated variants of the sequences encoding *UBP1 *without the transmebrane region. Using the PCR technique, two shortened genes were prepared without the 5' parts of the *UBP1 *coding region. Proteins encoded by these variants of the gene are shorter by 54 and 98 amino-acids (Figure 1). Expression of these analogues of the *UBP1 *gene inserted into pT7RS derivatives was analyzed. In addition, the influence of one mutation, named Q754L, within the *UBP1 *gene, which happened during PCR amplification, on the expression level was investigated (Figure 1A).

### Expression of the UBP1 variants in *E. coli*

**Figure 2:**
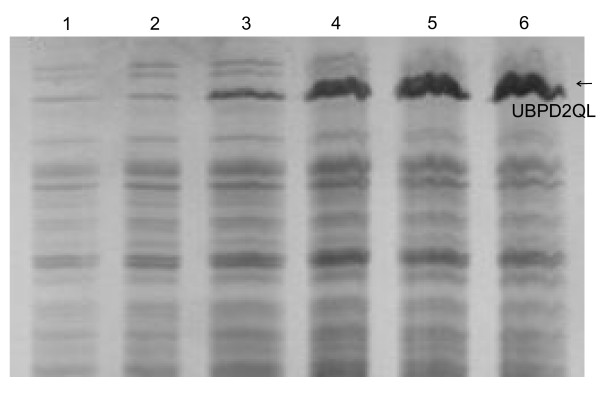
**Dependence of the expression level of the shorter analogue of UBP1, UBP1D2QL, on the time after IPTG induction as revealed by SDS electrophoresis in 12% polyacrylamide gel**. *E. coli *BL21 (DE3) cells transformed by the plasmid pT7UPD2QL were cultured at 25°C in LB, and induced by the addition of IPTG to the final concentration of 1 mM. Aliquots of cultures were taken at: 1 – 0 min, 2 – 30 min, 3 – 60 min, 4 – 90 min, 5 – 120 min, 6 – 150 min after induction.

**Figure 3:**
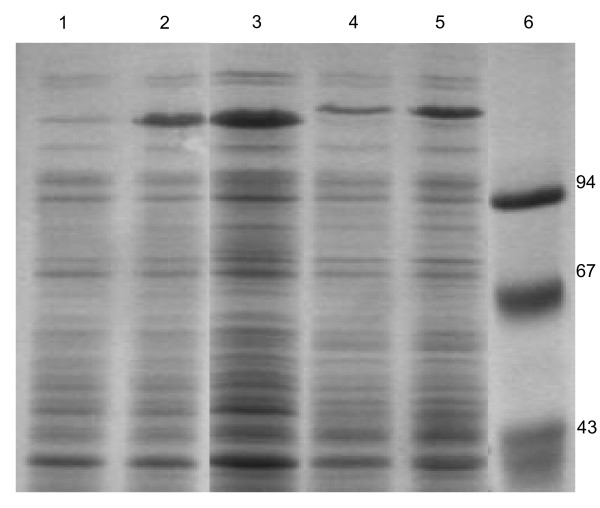
**Expression level of the *UBP1 *analogues as revealed by SDS electrophoresis in 12% polyacrylamide gel**. 1 and 2 – lysates of *E. coli *BLD3(DE3) bacteria transformed with pT7UPD2QL, not induced and induced with IPTG from cultures maintained in 100 ml LB media, respectively; 3 – supernatant after sonication of the bacteria culture performed in the fermenter; 4 and 5 – lysates of *E. coli *BLD3 bacteria transformed with pT7UPDQL, cultured, not induced and induced with IPTG, respectively.

**Figure 4:**
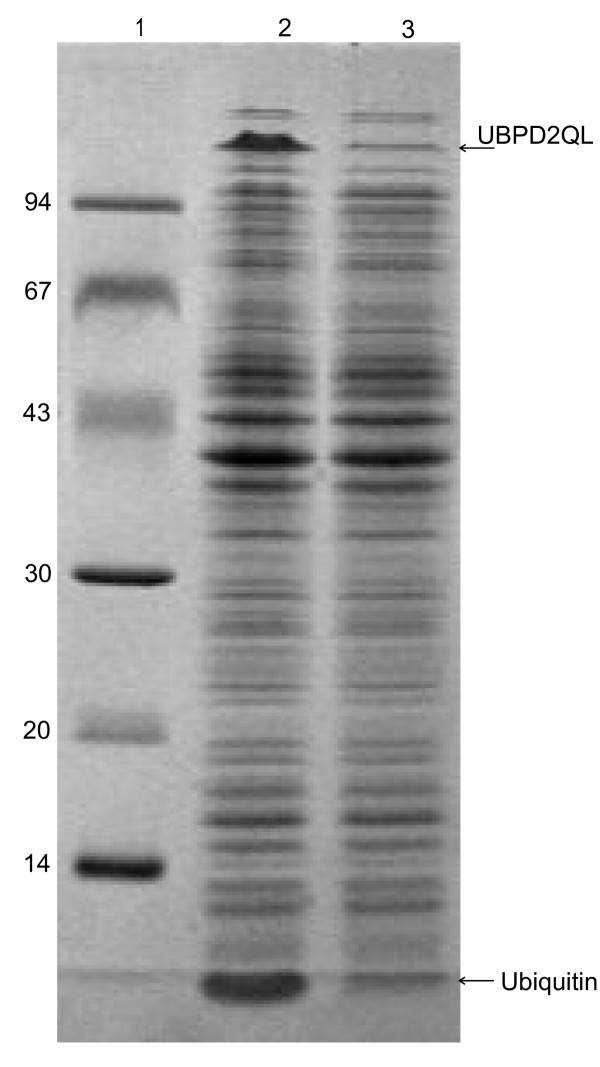
**Activity of UBPD2QL protease in bacterial cells, SDS electrophoresis in 12% polyacrylamide of bacterial lysates**. 1 – molecular mass marker (kDa); 2 and 3 – lysates of *E. coli *BLD3 (DE3) bacteria transformed with pT7UPDQL2, cultured, induced and not induced with IPTG, respectively. The ubiquitin band is marked with an arrow.

**Figure 5:**
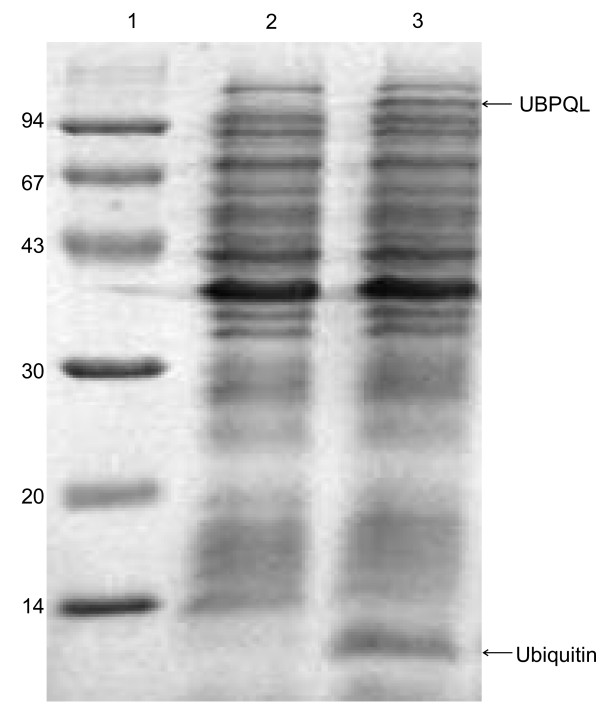
**Expression level of UBP1 analogue with Q754L mutation (UBPQL) as revealed by SDS electrophoresis in 12% polyacrylamide gel**. 1 – molecular mass marker (kDa); 2 and 3 – lysates *E. coli *BLD21(DE3) bacteria transformed with pT7UPQL, cultured, not induced and induced with IPTG, respectively. The UBPQL protease and ubiquitin bands are marked with arrows.

The obtained plasmids with the hybrid genes encoding the analogues: *UBI::UBPD*, *UBI::UBPDQL*, and *UBI::UBPD2QL *were used to transform the *E. coli *BLD21 strain. In general, several expression host strains and different culture conditions were examined (results not shown) in our mini-induction screening experiments to optimize recombinant protein expression. The cultures were grown at 37°C and 25°C until OD_600 nm _reached 0.5–1.8 and 1 ml samples were removed from each culture and saved as controls. Then, isopropyl β-_D_-thiogalactoside was added to the cultures at the desired concentration of 0.5–1.0 mM. Aliquots of cultures were taken 30, 60, 90, 120 and 150 min. post-induction (Figure 2). Cell density in these samples was measured to monitor the protein induction process and to normalize total proteins in *E. coli *after cell lysis in the 2xSDS sample buffer. The expression level (Figure 3) of the recombinant proteins was determined by a densitometric analysis of the electrophoretic pattern. The protein level was equal to 7.2 % and 9.8 % of the proteins visible in lines 2 and 5 (Figure 3) for UBPDQL and UBPD2QL, respectively. The results shown in Fig. 4 and 5 indicate that the fusions of ubiquitin with UBP1 analogues are active proteases during cell culture because an intensive band of ubiquitin is visible on SDS gels after electrophoresis of *E. coli *lysates.

The main factors influencing the expression level of *UBP1 *analogues and growth rate of bacteria are: the presence or absence of the Q754L mutation (Figure 5) and lack of the transmembrane region at the N-end of UBP1 (Figure 3). The lack of Q754L mutation in the *UBP1 *analogue gene makes an efficient production of the enzyme impossible because of a very slow culture growth rate and a low level of protease expression (results not shown). Apart from that we have shown that the removal of the transmembrane region leads to a significant reduction in growth time and an increase in the expression level (Figure 3). The period of time to reach OD_600 _= 1 for UBPD, UBPDQL and UBPD2QL was 48, 12 and 9 h, respectively. It appears also that the bacteria containing the vector with the shorter gene of the *UBP1 *analogue encoding *UBPD2QL *produce the highest amount of the recombinant protease (Figure 3 and 4). It was also established that the presence of the proper codons in the gene used for expression additionally shortened the period of time necessary to reach OD_600 _= 1 (results not shown). All things considered, the best expression system consists of the gene variant with the Q754L mutation, larger deletion of the N-end encoding sequence and proper codon usage. The best results were obtained when the bacteria culture was carried out at 25°C, the induction with IPTG begun when OD had reached 1 and when the culture time after induction was 2h (Figure 2).

### Purification of the recombinant UBPD2QLHisx6 protease and fusion proteins of the type 6xHisUBI::protein

**Figure 6:**
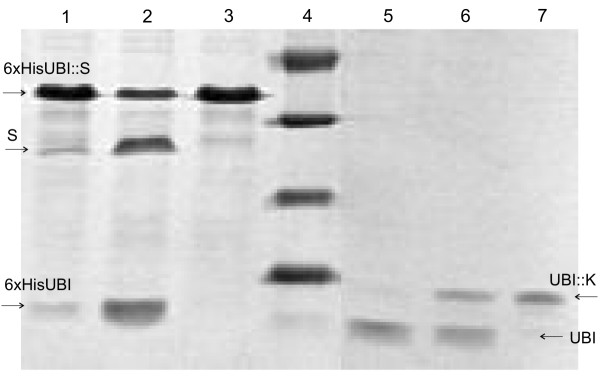
**Activity of the UBP1 analogues, UBPDQL and UBPD2QL, as revealed by SDS electrophoresis in 12% polyacrylamide gel**. 1 and 2 – the substrate 6xHisUBI::S digested with UBPDQL and UBPD2QL, respectively; 3 – the undigested substrate, 6xHisUBI::S; 4 – molecular mass marker (kDa); 5 and 6 – the substrate UBI::K digested with UBPD2QL and UBPDQL; 7 – the undigested substrate, UBI::K.

For purification, the cells from 0.5 litre culture after a 2-hour induction at 25°C in the presence of 1 mM IPTG were harvested. The pellet was resuspended in 50 ml of buffer A. All subsequent steps were performed at 4°C. The cells were disrupted by sonication on ice and the insoluble debris was removed by centrifugation for 25 min. at 11500 rpm. The cleared extract was chromatographed on a Ni-NTA Superflow column, pre-equilibrated with 10 vol. of buffer A. After loading, the column was washed with 5 vol. of buffer B and then the protease was eluted twice with elution buffer C. Protein concentrations were determined using the Bradford dye binding assay and bovine serum albumin as the standard. Both analogues of UBP1, UBPDQL and UBPD2QL, were purified in the same manner. Digestion of the two substrates, 6xHisUBI::S and UBI::K, shows that the preparations of the two different UBP1 analogues are active proteases, but the activity of the shorter one (UBP1DC2) is higher (Figure 6). Stability of both the UBPDQL and UBPD2QL analogues during long exposure to 37°C was also investigated. It appeared that both enzymes were active after 24 h incubation (results not shown).

We obtained 13.8 mg of purified UBPDQL and 20 mg of purified UBPD2QL from 1 l of *E. coli *culture. The amounts represent 6.3% and 7.4% of the total protein obtained after the destruction of bacterial cells, and correspond to 710 U of the purified UBPDQL and 1650 U of UBPD2QL, respectively.

**Figure 7:**
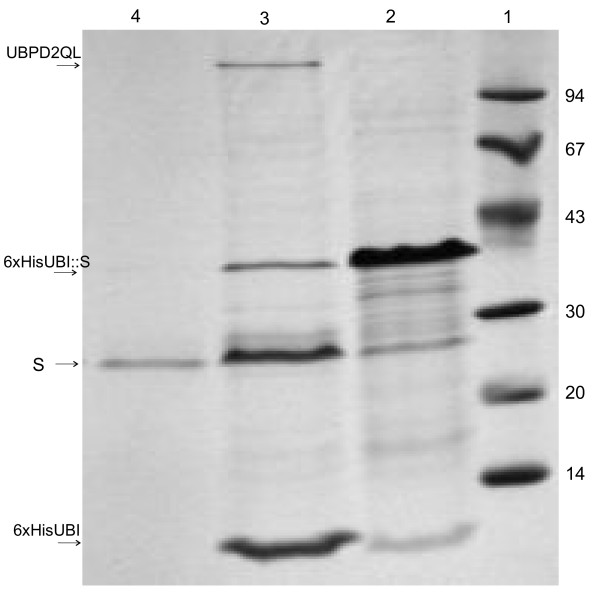
**Digestion of the 6xHisUBI::S fusion with UBPD2QL and purification of peptide S on an Ni-NTA column. SDS electrophoresis in 12% polyacrylamide gel**. 1 – molecular mass marker (kDa), 2 – 6HisUbi::S purified in a chromatography column containing Ni-NTA medium, 3 – 6xHisUBI::S digested with protease UBPD2QL, 4 – S protein purified in a chromatography column filled with Ni-NTA medium.

We would like to stress that the presence of 6xHis at the C-end of UBP1 analogues may greatly facilitate purification of the recombinant protein containing ubiquitin tagged at its N-end with 6 histidine residues. We propose a three steps procedure: 1) purification of the fusion protein on a Ni-NTA column followed by dialysis; 2) digestion of the purified fusion with a UBP1 analogue, and 3) chromatography of the digestion mixture on the same column. The protein 6xHisUBI::S, used for activity determination, was treated in this way (Figure 7). SDS-PAGE image analysis showed that this simple procedure leads to an almost pure peptide because of the undigested fusion protein. His-tagged ubiquitin and protease bind to the Ni-NTA column while after the digestion of the fusion, the liberated protein flows out of the Ni-NTA column. Such an approach greatly simplifies the purification of the fusion proteins and the limited number of purification stages greatly enhances the efficiency of the whole process.

It should be noted that a solution similar to ours, produced artificially as a result of UBP1 gene truncation [6], was observed by Schmitz et al. [7], who discovered two forms of UBP1 in the yeast cells, the anchored and the soluble one.

## Conclusion

Our results show that an efficient expression of the unmodified yeast *UBP1 *protease gene in *E. coli *in the presented expression system is impossible. The most important change to be introduced into the *UBP1 *gene is the Q754L mutation leading to the replacement of glutamine by leucine at position 754 in the aa sequence in the yeast UBP1. The removal of the transmembrane N-end region of *UBP1 *improves the level of expression of a properly truncated gene. Both of these changes in the gene decrease the time needed for bacteria cultivation.

We have shown that using protease analogues, tagged at the C-end by 6xHis, for cleaving fusion proteins containing N-end His-tagged ubiqutin of the type 6xHisUBI::protein facilitates purification of the protein present in the hybrid to a great extent.

The high expression level in *E. coli *of our *UBP1 *analogues allows the use of ubiquitin::protein fusion in large scale production of recombinant proteins.

## Methods

### Bacterial Strains, Plasmids, Enzymes, and Reagents

*Saccharomyces cerevisiae*, strain W303, was used as a source of the DNA for PCR amplification. The *E. coli *DH5α, NM522 strains were used for transformations to obtain recombinant plasmids. The *E. coli *BLD21 (DE3) strain was applied to achieve expression of the His6-tagged analogues of the UBP1 protease. The plasmid pT7RS (GenBank Accession No. AY923866), containing ArgU tRNA (UCU) gene and the transcription terminator of phage T7 RNA polymerase, was used for the construction of the expression system. The *E. coli *BLD21 (DE3) cells with the pT7RS plasmid derivatives were cultured aerobically at 25°C or 37°C in LB medium supplemented with 50 μg/ml ampicilin. Restriction, modification enzymes were purchased from Amersham Pharmacia Biotech. The reagents for PCR and Ni-NTA Superflow columns were obtained from Qiagen. IPTG, agarose, polyacrylamide and the reagents for protein purification were purchased from Sigma-Aldrich ChemieGmbH, Steinheim, Germany. For site-directed mutagenesis the Stratagene kit was used (Cat. No. 200518-5). We used the GelScan v. 1.45 software package for the densitometric analysis of the electrophoretic image.

The buffers used for the purification of UBP1 analogues were: buffer A (50 mM buffer phos. pH 8.0; 0.3 M NaCl; 10 mM imidazol; 10 mM 2-β-mercapthoethanol; 10% glicerol; 0,1% Triton), buffer B (50 mM phosphate buffer, pH 8.0; 0.3 M NaCl; 40 mM imidazol; 10 mM 2-β-mercapthoethanol; 10% glicerol; 0,1% Triton) and buffer C (50 mM phosphate buffer pH 8.0; 0,3 M NaCl; 300 mM imidazol; 10 mM 2-β-mercapthoethanol; 10% glicerol; 0,1% Triton).

### Plasmid construction

To facilitate subsequent purification of the UBP1 variants or fusion proteins, the pT7CH, pT7NH, pT7U and pT7NHU plasmids were constructed. The first one contains in the 3' polilinker part the 6 histidines coding sequences followed by a TAA stop triplet. The second one was constructed for the addition of 6 histidines tags to the N-ends of hybrid proteins. It contains 6 histidines coding sequences following an ATG triplet.

The pT7CH was obtained by inserting a short double stranded DNA fragment formed by two synthetic oligonucleotides, HIST7G and HIST7D (Table 1), into the pT7RS plasmid digested with *Eco*RI and *Hind*III restriction nucleases. Similarly, the pT7NH was constructed by inserting a double stranded DNA fragment formed by *6HisG *and *6HisD *oligonucleotides (Table 1) into pT7RS digested with *Nde*I and *Eco*RI.

**Table 1:** Primers for the construction of pT7RS and *UBP1 *derivatives

**Primers**^a^	**Sequences**^b^	**Restriction site**
HIST7G	5' AATTCGATATCGTCGACGGATCC**CATCATCACCATCACCAT**TAAAAT 3'	*Eco*RI
HIST7D	5' AGCTATTTTAATGGTGATGGTGATGATGGGATCCGTCGACGATATCG 3'	*Bam*HI, *Sal*I, *Eco*RV,
6HisG	5' TATGGCA**CATCATCACCATCACCAC**TCTGGTTCTG 3'	*Nde*I
6HisD	5' AATTCAGAACCAGAGTGGTGATGGTGATGATGTGCCA 3'	*Eco*RI
UB1G	5' GGGGAATTCATATGCAGATTTTCGTCAAAACTTTG 3'	*EcoR*I and *Nde*I
UBID2	5' GGGGATCCTTAATGCTCTTCACCACCGCGGAGTCTTAAG 3'	*Bam*HI and *Sac*II
UBP1G	5' AGACTCCGCGGTGGTGATTTGTTTATTGAAAGCAAGATA 3'	*Sac*II
UBP1D	5' GGGGATCCTTAGTTTACATCTTTACCAGAAATA 3'	*Bam*HI
UBP1MG	5'GGCATAGTAGTATTTTTTTACCGCGGTGGTGACCATCTAAACTACATTGT 3'	*Sac*II
UBP1MD	5'ACAATGTAGTTTAGATGGTCACCACCGCGGTAAAAAAATACTACTATGCC 3'	*Sac*II
SkrutG	5'AAAACCGCGGTGGTTTCATTGCTGGTTTA 3'	*Sac*II
SkrutD	5'GGAAGAATTCTTGCGCGTCCTC 3'	*Eco*RI
UBP1GC	5'-GATTTGGAAGCTATT**CAG**AGTAATAATGAAG-3'	
UBP1DC	5'-CTTCATTATTACTCTGAATAGCTTCCAAATC-3'	
KalaG	5'-GATCCAGGCGATAAAGACGGTGATGGCTATATTTCTGCTGCTGAAGCTATGGCTTA-3'	*Bam*HI
KalaD	5'-AGCTTAAGCCATAGCTTCAGCAGCAGAAATATAGCCATCACCGTCTTTATCGCCTG-3'	*Hind*III

^a ^The names of primers contain the letters: G – sense or D – antisense.

^b ^The restriction sites are underlined or overlined. The histidine and leucine codons are in bold face.

The pT7U plasmid contains a nucleotide sequence encoding modified yeast ubiqutin with the *Sac*II restriction nuclease recognition sequence near the 3' end of the sequence, facilitating construction of different hybrid genes. Primers UB1G and UBID2 (Table 1) were used to amplify and modify the ubiquitin gene. The plasmid was used to express the fusion proteins: ubiquitin::modified UBP1.

The pT7NHU plasmid contains a synthetic nucleotide sequence encoding ubiquitin with the codons used most frequently in the *E. coli *genome, inserted into the pT7NH plasmid. This plasmid was used to express a hybrid gene for the synthesis of the substrate for the determination of UBP1 activity.

The *UBP1 *protease gene was obtained using PCR. For amplification, the UBP1G and UBP1D primers were used (Table 1). The oligonucleotides contained recognition sites for the restriction endonucleases *Sac*II and *Bam*HI, respectively. The PCR was performed in a 50 μl reaction volume with a buffer containing 50 mM KCl, 1.75 mM MgCl_2_, 0.02 mM of each dNTP, 10 mM Tris-HCl (pH 8.9), 100 pM of each primer, the enzyme mixture of *Taq *and *Pwo *DNA polymerases (Expand Long Template PCR System, Roche) and 100 ng of total DNA of *S. cerevisiae *as a template for 30 cycles using Eppendorf 5330 thermocycler. Each cycle consisted of 15 sec at 94°C, 15 sec at 56°C, and 2 min at 72°C. The amplified 2430-bp-long DNA fragment was isolated by 1% agarose gel electrophoresis using Gel-Out kit (Kucharczyk T. E. Co, Warsaw), digested with *Bam*HI and *Sac*II restriction nucleases, purified and ligated to *Bam*HI and *Sac*II digested pT7U vector. Ligation products were transformed into the NM522 *E. coli *strain. Plasmid DNA was isolated using the alkaline method [8]. Next, the 2430 bp *UBP1 *gene was excised from the recombinant plasmid using the restriction enzymes *Nde*I and *Bam*HI, and the DNA fragment encoding hybrid gene *ubiquitin::UBP1 *was recloned into the pT7CH plasmid at the *Nde*I and *Bam*HI sites. In this way the pT7UPQL vector was obtained (Table 2).

**Table 2:** Plasmids and genes used to construct vectors of the pT7RS derivatives

**Vectors**	**Gene cloned**	**Result ing vectors**	**Expression products**	**Name of the protein used in the text**
pT7SR	*Ubiquitin*	pT7U	Ubiquitin	UBI
pT7U	*K*	pT7UK	UBI::K	UBI::K
	*UBP1*	pT7UP	UBI::UBP1	UBP1
pT7CH	*UBI::UBP1 *with Q754L mutation	pT7UPQL	UBI::UBPQL::6xHis	UBPQL
	*UBI::UBP1(55-809) *without Q754L mutation	pT7UPD	UBI::UBPD::6xHis	UBPD
	*UBI::UBP1(55-809) *with Q754L mutation	pT7UPDQL	UBI::UBPDQL::6xHis	UBPDQL
pT7CH	*UBI::UBP1(99-809) *with Q754L mutation	pT7UPD2QL	UBI::UBPD2QL::6xHis	UBPD2QL
pT7NH	*Ubiquitin*	pT7NHU	6xHis::UBI	6xHisUBI
pT7NHU	*S*	pT7NHUS	6xHis::UBI::S	6xHisUBI::S

### UBP1 variants

PCR was used to remove the transmembrane domain from the *UBP1 *gene. Two variants were obtained. In the first variant, a 162 bp fragment was removed from the 5' part of the coding sequence. For this purpose, site-directed mutagenesis was used with the primers UBP1MG and UBP1MD (Table 1). The shortened gene was modified by the addition of '5-GGTGGT-3', the sequence encoding Gly-Gly, the C-end amino-acids of ubiquitin, and the *Sac*II nuclease recognition sequence. We called this mutein UBPDQL (Figure 1).

The second, shorter variant of *UBP1 *was prepared by PCR using SkrutG and SkrutD primers (Table 1). In this way an additional 132 bp long DNA fragment was removed. The new variant of the protease consisted of 711 aa (Figure 1). We named this protease variant UBPD2QL. The two shorter variants of the *UBP1 *gene were inserted into the pT7UCH expression plasmid. The obtained plasmids were designated pT7UPDQL and pT7UPD2QL, and used for the synthesis of UBPDQL and UBPD2QL proteases, respectively (Table 2).

Both variants of the modified gene contain the same mutation leading to CAG to CTG codon change (gln → leu, Figure 1A), which appeared after the first amplification of the *UBP1 *gene. This mutation was removed using the site-directed kit with the primers UBP1GC and UBP1DC (Table 1).

To circumvent the codon usage problem [9], the *UBP1 *protease gene was modified through the exchange of certain argining codons (AGA or AGG for CGT or CGC) and leucine codons (TTA for CTG). Stratagene mutagenesis kit with *turbo*-polymerase was used for site-directed mutagenesis. In this way the following replacements were made: arginine's codons in positions 96, 334, 425, 476, 482, 487, 613, 702, 705, 710, 796 and 801, and leucine codons in positions 354, 356, 358, 367, 369, 372, 373, 469 and 470 of the UBP1 amino-acid sequence (Figure 1A).

### Determination of the activity of the UBI::UBP1 protease variants

In order to determine the protease activity of the UBP1 analogues, two hybrid proteins were obtained. To obtain the first one, the 354 bp long DNA fragment, encoding the C-terminal end of the UBP1 protease named *S*, was cloned into the pT7NHU plasmid (Figure 1A, B). The second one consists of yeast ubiquitin followed by: AspProGlyAspLysAspGlyAspGlyTyrIleSerAlaAlaGluAlaMetAla-, a peptide analogous to the IIId calcium-binding loop of calmodulin [10]. The sequence encoding this fusion peptide was obtained by ligation of the yeast ubiquitin gene with synthetic oligonucleotides: KalaG and KalaD (Table 1), and cloned into pT7U. Both plasmids were used to transform *E. coli *BLD21 (DE3) cells with the aim of obtaining the hybrid proteins 6xHisUBI::S and UBI::K (Table 2). Both fusion proteins were soluble during the synthesis in *E. coli*. The 6xHisUBI::S was purified by Ni-NTA affinity chromatographs. The UBI::K protein was purified by ion exchange chromatography on a column of DEAE-Sepharose Fast Flow (Pharmacia LKB), followed by NiCl affinity chromatography using Chelating Sepharose Fast Flow (Pharmacia LKB). In both cases, the expression level and purity of the hybrid proteins were high enough (data not shown) to be used for activity determination. The reactions were performed in a volume of 50 μl at 37°C for 30 min. in a buffer of the following composition: 20 mM phosphate pH 7.5, 2 mM DDT, 1 mM EDTA. 4 μg (380 pM) of the substrate UBI::K or 2 μg (87 pM) of 6xHisUBI::S were digested with 1.5 μg (18.2 pM) of the protease variants. The digestion reactions were stopped by heating at 100°C for 3 min. in the presence of SDS, and the digestion products were analyzed using SDS-PAGE (12%) (Figure 6). The unit (U) of enzyme activity is defined as the amount (of the enzyme) which will catalyze the transformation of 1 micromole of the substrate per minute under standard conditions.

## Abbreviations used

aa – amino-acid residue

bp – base pair(s)

IPTG – isopropyl-β-D-galactoside

kDa – kilodalton

LB – Luria-Bertani

PCR – polymerase chain reaction

U – unit

UBI – ubiquitin

UBP – ubiquitin-specific protease (Ubp)

SDS – sodium dodecyl sulfate

SDS-PAGE – sodium dodecyl sulfate-polyacrylamide gel electrophoresis

## Authors' contributions

AW carried out the molecular genetics studies, participated in the purification of the proteins and drafted the manuscript. AMP carried out the purification of the UBP1 analogues and helped to draft the manuscript. DMS participated in the construction of the *UBPDQL *gene. GP was involved in the purification of the UBPDQL protease. LCh constructed the synthetic gene of ubiquitin. NŁ was involved in the purification of the UBI::K protein. The study was conceived and coordinated by AP, who also helped to draft the manuscript.

All the authors read and approved the final manuscript.
